# Characterization of innate and adaptive immune cells involved in the foreign body reaction to polypropylene meshes in the human abdomen

**DOI:** 10.1007/s10029-021-02396-7

**Published:** 2021-03-31

**Authors:** A. Dievernich, P. Achenbach, L. Davies, U. Klinge

**Affiliations:** 1 Department of General, Visceral and Transplant Surgery, RWTH Aachen University Hospital, Pauwelsstraße 30, 52074 Aachen, Germany; 2 Institute of Neuropathology, RWTH Aachen University Hospital, Aachen, Germany; 3 Division of Infection and Immunity, Cardiff University, Cardiff, UK

**Keywords:** Foreign body reaction, Adaptive immune system, Innate immune system, Mesh, Fluorescence microscopy

## Abstract

**Background:**

Polypropylene (PP) mesh is widely used to reinforce tissues. The foreign body reaction (FBR) to the implant is dominated by innate immune cells, especially macrophages. However, considerable numbers of adaptive immune cells, namely T cells, have also been regularly observed, which appear to play a crucial role in the long-term host response.

**Methods:**

This study investigated the FBR to seven human PP meshes, which were removed from the abdomen for recurrence after a median of one year. Using immunofluorescence microscopy, the FBR was examined for various innate (CD11b^+^ myeloid, CD68^+^ macrophages, CD56^+^ NK) and adaptive immune cells (CD3^+^ T, CD4^+^ T-helper, CD8^+^ cytotoxic, FoxP3^+^ T-regulatory, CD20^+^ B) as well as “conventional” immune cells (defined as cells expressing their specific immune cell marker without co-expressing CD68).

**Results:**

T-helper cells (19%) and regulatory T-cells (25%) were present at comparable rates to macrophages, and clustered significantly toward the mesh fibers. For all cell types the lowest proportions of “conventional” cells (< 60%) were observed at the mesh–tissue interface, but increased considerably at about 50–100 µm, indicating reduced stimulation with rising distance to the mesh fibers.

**Conclusion:**

Both innate and adaptive immune cells participate in the chronic FBR to PP meshes with T cells and macrophages being the predominant cell types, respectively. In concordance with the previous data, many cells presented a “hybrid” pattern near the mesh fibers. The complexity of the immune reaction seen within the foreign body granuloma may explain why approaches focusing on specific cell types have not been very successful in reducing the chronic FBR.

**Supplementary Information:**

The online version contains supplementary material available at 10.1007/s10029-021-02396-7.

## Introduction

Polypropylene (PP) meshes are widely used in surgery to reinforce tissues, in particular for the treatment of abdominal wall hernia or pelvic floor instability. After implantation of biomaterials, blood/material interactions occur first, followed by migration of immune cells marking the onset of inflammation, development of granulation tissue, and remodeling with formation of a fibrous capsule that shields the foreign body [1, 2]. The tissues reaction to the implant is dominated by cells of the innate immune system, especially CD68^+^ macrophages [3]. The morphologic structure consisting of cellular infiltrate and fibrotic capsule is denoted foreign body granuloma (FBG). Owing to their persistence at the mesh–tissue interface, macrophages are held responsible for the chronic inflammatory process. However, considerable numbers of lymphocytes have also been regularly observed in the FBG [4].

Granulomatous inflammation is a hallmark in several infectious (e.g., mycobacterial) and noninfectious (e.g., sarcoidosis, Crohn’s disease) diseases in response to persistent stimuli [5–8]. The central part of the granulomas usually consists of various macrophage populations and sparse multinucleated giant cells surrounded by a lymphatic cuff of T- and B-cells as well as fibroblasts, with mature granulomas also featuring a fibrotic layer [5, 6, 9–11]. However, the architecture and immunology of granuloma formation differ significantly between granulomatous disorders [12]. The FBG is considered the most basic form of granulomatous inflammation, which fundamentally differs from granulomatous diseases because foreign bodies do not prominently feature adaptive immune responses [12, 13].

Classically, host immunity is separated into the innate (fast and unspecific) immunity mediated by myeloid cells, such as macrophages and natural killer (NK) cells, and the adaptive (slower but specific) immunity based on B- and T-lymphocytes [14]. Although Mills et al. already postulated in 2000, a collaboration between macrophages and lymphocytes, innate, and adaptive immune processes were long regarded as separate or relatively compartmentalized in time [15]. However, research over the last decades has clearly demonstrated strong links between both immune systems [14].

In 2019, Tennyson et al. pointed out that cells of the adaptive immune system, namely T-helper cells (CD4^+^), cytotoxic T-cells (CD8^+^), and regulatory T-cells (FoxP3^+^), appear to play a crucial role in the long-term host response to pelvic floor meshes and remain elevated in the FBG years after implantation [16]. Similar results were observed by Artsen et al., although with different implications regarding regulatory T-cells in relation to fibrosis [17]. Although Tennyson et al. suggested that T-helper cells and regulatory T-cells promote a fibrotic response, Artsen et al. concluded that the latter rather have an antifibrotic effect. Obviously, the role of lymphocytes in the foreign body reaction (FBR) to mesh is still matter of debate. Moreover, a possible importance of the adaptive immune system after implantation of PP meshes is underlined by the observations of Cohen Tervaert in 40 patients who developed symptoms of a systemic illness [18]. After complete mesh removal in 6 patients, they recovered (partially), leading the author to postulate that PP mesh as an adjuvant may increase the risk of developing lymphocyte related (auto)immune diseases.

Previously, we reported that CD68 is co-expressed on various cell types participating in the FBR to meshes, which is considered a “hybrid” pattern in contrast to “conventional” cells expressing their specific surface markers without CD68 [19]. Subsequently, we showed that tissue remodeling macrophages dominate at the interface of fibrotic PP meshes in the human abdomen that have been removed for the primary complaint of recurrence [20].

The aim of this study was to further characterize the chronic FBR to the PP meshes, which were removed for recurrence. We analyzed the presence of several innate (CD11b^+^ myeloid cells, CD68^+^ macrophages, CD56^+^ NK cells) and adaptive (CD3^+^ T lymphocytes, CD4^+^ T-helper cells, CD8^+^ cytotoxic T-cells, FoxP3^+^ regulatory T-cells, CD20^+^ B lymphocytes) immune cells using immunofluorescence microscopy. We examined the total number and percentages of cells located within a 1 mm^2^ circular region of interest placed around mesh fibers and their spatial distributions using distance maps. Finally, we looked for the proportions of “conventional” immune cells (defined as cells not co-expressing CD68) relative to the total number of positive cells for each cell type as an indicator of the sustained mesh-induced stimulus.

## Materials and methods

**Fig. 1:**
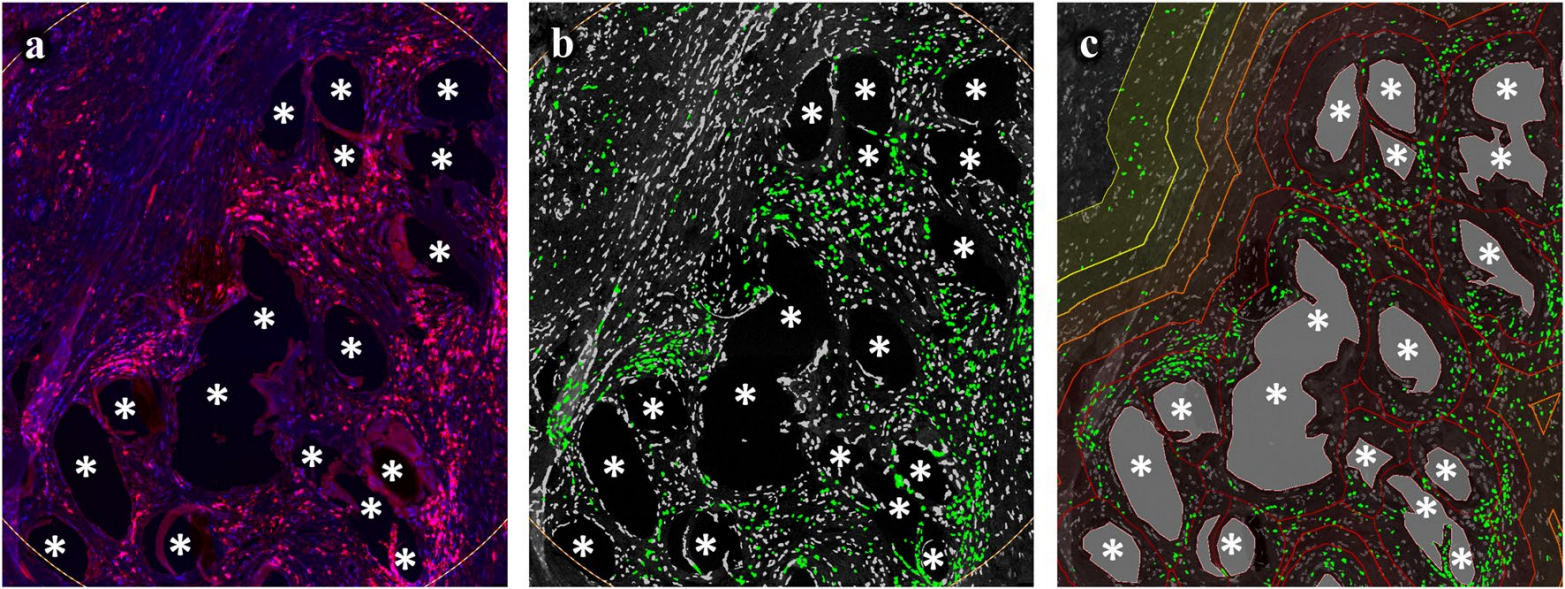
Immunofluorescence labeling of T cells (CD3) with region of interest (ROI) and distance map analysis. **a** Labeling of T cells with Cy5 (red) and nuclei with DAPI (blue). **b** Backward gating of CD3^+^ T cells (green) using ROI analysis. **c** Backward gating of CD3^+^ T cells (green) with superimposed distance map. Backward gating is always on the nuclei shade. The Euclidean distance map consists of six regional zones from 0–50 µm (dark red) to 250–350 µm (bright yellow) in 50 µm steps. Locations of mesh fibers are marked with asterisks, scale bar = 100 µm. Images of explant #3 (color figure online)

**Fig. 2:**
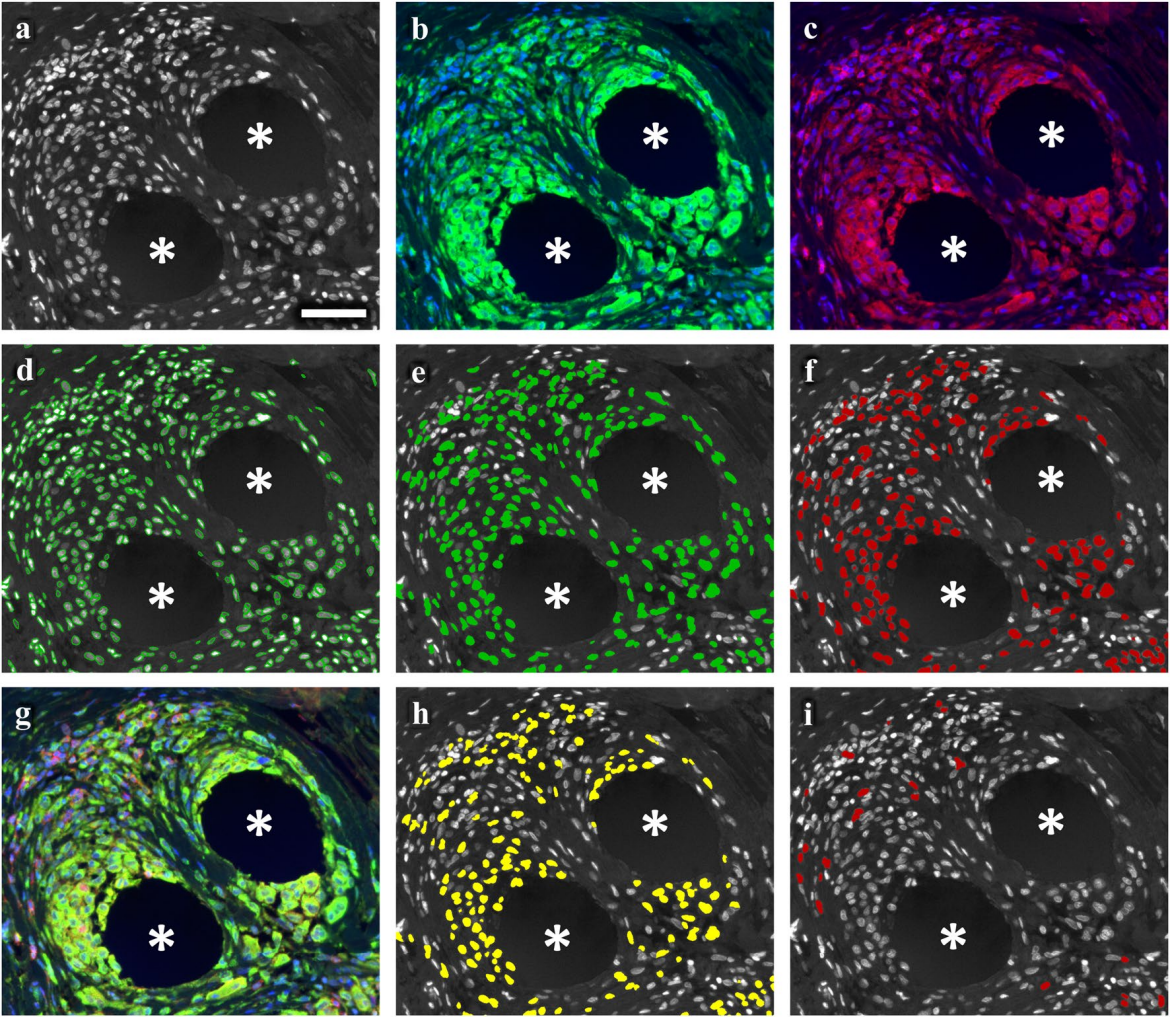
Nuclear detection and backward gating of immune cells participating in the foreign body reaction. Immunofluorescence labeling of pan-macrophage marker CD68 (FITC), CD4 (Cy5), and nuclei (DAPI). **a** Grayscale nuclei shade. **b** CD68 (green) and nuclei (blue). **c** CD4 (red) and nuclei (blue). **d** Backward gating of detected nuclei (green contours). **e** Backward gating of CD68^+^ cells (green). **f** Backward gating of CD4^+^ cells (red). **g** Superposition of CD68 (green), CD4 (red) and nuclei (blue). **h** Backward gating of “hybrid” CD4^+^CD68^+^ cells (yellow). **i** Backward gating of “conventional” CD4^+^CD68^−^ cells (red). Backward gating is always on the nuclei shade. Locations of mesh fibers are marked with asterisks, scale bar = 50 µm. Images of explant #3 (color figure online)

**Fig. 3:**
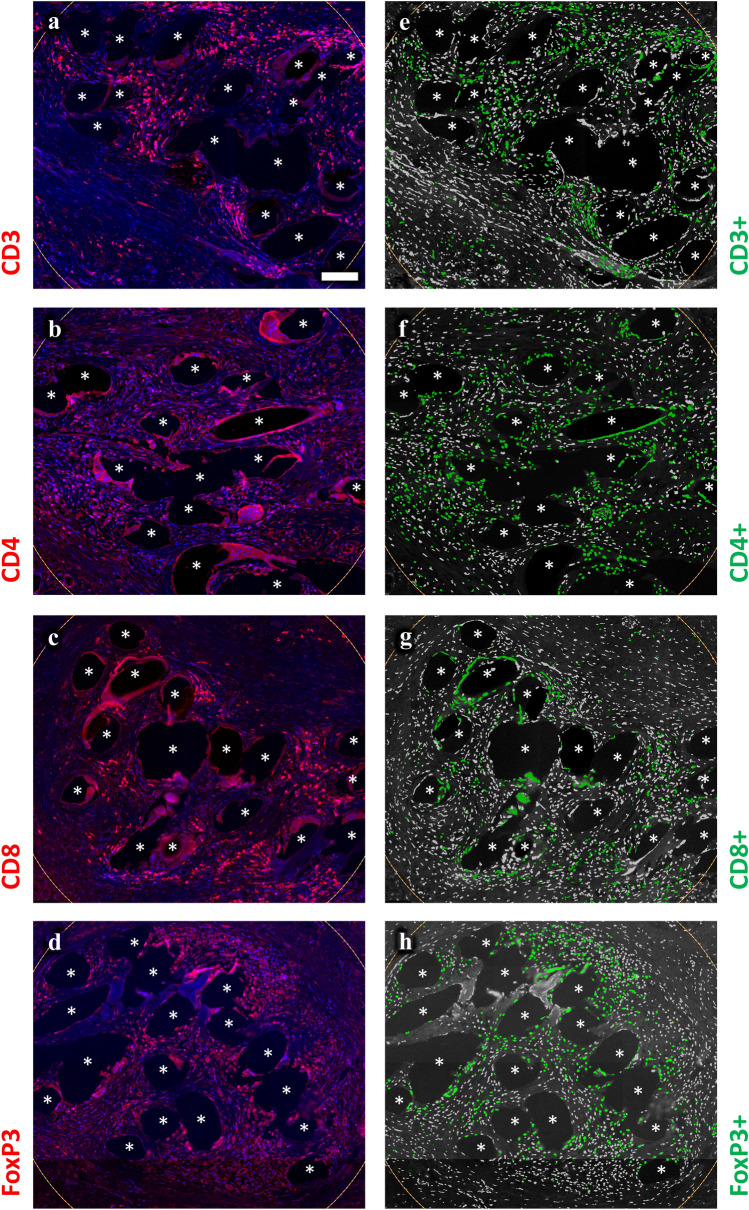
Region of interest selection and immunofluorescence labeling of immune cells part1. Labeling of T cells (CD3), T_h_ (CD4), T_c_ (CD8), and T_regs_ (CD8). Nuclei are labeled with DAPI (blue). **a**–**d** Labeling of cells with Cy5 (red) and **e–h** backward gating of “positive” cells (green), respectively. Cells with a mean staining intensity > 100 are considered to be “positive”. Locations of mesh fibers are marked with asterisks, scale bar = 100 µm. Images of explants #7 (color figure online)

**Fig. 4:**
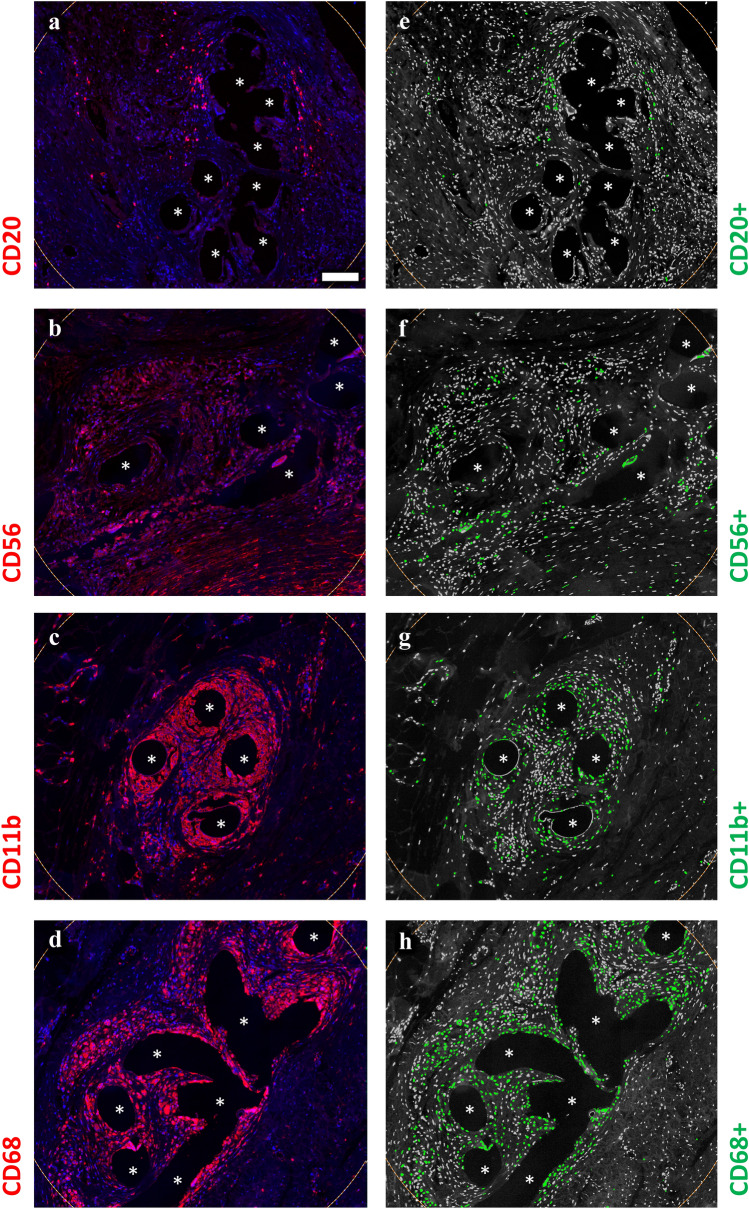
Region of interest selection and immunofluorescence labeling of immune cells part2. Labeling of B cells (CD20), NK cells (CD56), myeloid cells (CD11b), and macrophages (CD68). Nuclei are labeled with DAPI (blue). **a**–**d** Labeling of cells with Cy5 (red) and **e**–**h** backward gating of “positive” cells (green), respectively. Cells with a mean staining intensity > 100 are considered to be “positive”. Locations of mesh fibers are marked with asterisks, scale bar = 100 µm. (**a**, **e**) Images of explant #6, (**b**, **f**) images of explant #2, (**c**, **d**, **g**, **h**) images of explant #3 (color figure online)

We analyzed new tissue sections from seven polypropylene (PP) meshes that were used for abdominal wall hernia repair; and have been explanted for recurrence after a median incorporation time of 1 year. Medical records were reviewed, and the following meshes were recorded: 3 large pore Ultrapro^®^, 2 small pore plugs, and 2 Ventralex^®^ with a layer of PTFE (Table 1). To minimize the influence of the PTFE layer of Ventralex^®^ meshes, the layer and adjacent cells were spatially excluded (s-Fig. 1). Human tissue samples of liver, lymph node, tonsil, and spleen without gross pathology served as healthy control tissues; and were used to check specificity of labeling and optimize antibody dilutions (s-Fig. 2–4; s-Table 1).

**Table 1:** Information on the patients whose mesh samples were examined

Explant no	Mesh type	Incorporation time [years]	Gender	Age
#1	Ultrapro^®^	0.6	Female	52
#2	Ultrapro^®^	0.9	Female	34
#3	Ultrapro^®^	1.3	Female	50
#4	Ventralex^®^	1.0	Male	47
#5	Ventralex^®^	6.0	Female	57
#6	Plug	2.2	Male	69
#7	Plug	4.0	Male	51

Prior to immunofluorescence staining, mesh samples were checked for the presence of characteristic cells and morphology by hematoxylin and eosin. All mesh–tissue complexes demonstrated a typical, highly localized foreign body reaction around the mesh fibers, consisting of a dense inner cellular infiltrate (predominantly mononuclear cells) surrounded by an outer fibrotic capsule. Morphologically, cellular infiltrates at the mesh–tissue interface always presented a mixture of mainly macrophages and lymphocytes with a few multinucleated foreign body giant cells.

### Immunofluorescent staining

The staining protocol used for this investigation has already been described in detail before [20]. Briefly, serial 2 µm sections of each specimen were double labeled with monoclonal antibodies (Table 2). The order of fluorophores was always kept constant. Tissue sections were deparaffinized, rehydrated before incubation in formalin. Then, antigens were retrieved. Afterwards, sections were washed and cooled before nonspecific binding was blocked. The pan-macrophage marker CD68 was applied and labeled with fluorescein isothiocyanate (FITC). Afterwards, tissue sections were microwave treated for antibody stripping and incubated overnight. The second marker was applied the following day and labeled with cyanine-5 (Cy5). The list of second markers includes CD3 [T lymphocytes (T cells)], CD4 [T-helper cells (T_h_)], CD8 [cytotoxic T-cells (T_c_)], CD11b (myeloid cells), CD20 [B lymphocytes (B cells)], CD56 [natural killer cells (NK cells)], and FoxP3 [regulatory T-cells (T_regs_)]. Finally, tissue sections were rinsed, counterstained with DAPI, and coverslipped.

**Table 2:** List of monoclonal antibodies used in this study sorted alphabetically

Antibody	Clone	Dilution	Incubation time	Manufacturer
CD3	F7.2.38	1:1000	30 min	Dako
CD4	4B12	1:500	30 min	Dako
CD8	CD8/144B	1:500	30 min	Dako
CD11b	OTI12C10	1:2000	30 min	OriGene
CD20	L26	1:200	30 min	Dako
CD56	123C3	1:200	30 min	Dako
CD68	KP1	1:6000	30 min	Dako
FoxP3	PCH101	1:250	30 min	eBioscience

### Analysis of fluorescence images/stainings

Fluorescence imaging was performed with an Axio Imager 2 epifluorescence microscope (20×, Zeiss, Germany) and the TissueFAXS PLUS system (TissueGnostics, Austria). Images were processed and quantitatively analyzed with StrataQuest Analysis Software (v6, TissueGnostics). We performed two separate analysis, the first with selected 1 mm^2^ circular regions of interest (ROIs) that were placed around mesh fibers and the second with manually outlined individual mesh fiber areas that were processed using Euclidean distance to establish distance maps with six regional zones (zone1: 0–50 µm, zone2: 50–100 µm, zone3: 100–150 µm, zone4: 150–200 µm, zone5: 200–250 µm, zone6: 250–350 µm) from the mesh fibers (Fig. 1). A median of 10 (range 5–28) ROIs and 35 (range 17–86) individual mesh fiber areas were analyzed. The detection of cells and positive marker signals was done as described previously [19]. Briefly, optimized DAPI images were used to detect and segment nuclei whose areas were used to measure the mean staining intensities for FITC- and Cy5-shades of the respective markers. Cells with a mean staining intensity above 100 were considered “positive” and detection was verified with backward gating (Fig. 2). We recorded the total number of nuclei, as well as the percentages of FITC^+^ (CD68^+^), Cy5^+^ (second marker^+^), and Cy5^+^FITC^−^ (second marker^+^CD68^−^) cells.

In addition, controls without primary antibody and controls with isotype antibodies were performed. The omission of the primary antibodies and substitution of the primary antibodies with the isotype antibodies at the same final concentrations resulted in lack of immunostaining.

### Statistical analysis

Calculations were done with MATLAB^®^ 9.1 and Image Processing Toolbox 9.5 (The MathWorks, US). Statistical analysis was performed with Statistical Package for Social Sciences software (SPSS^®^ v23, IBM, US). Statistical significance between different zones were determined with Mann–Whitney *U* test, *p* values < 0.05 were considered to be statistically significant.

## Results

### Innate and adaptive immune cells co-exist within the foreign body granuloma

By using 1 mm^2^ circular regions of interest (ROIs) placed around mesh fibers, we studied the cell densities, defined as cells per mm^2^ as well as the percentages of innate (macrophages, NK cells, CD11b^+^ cells) and adaptive (T cells, T_h_, T_c_, T_regs_, B cells) immune cells (as fraction of total cells). Within the ROI the cell density varied between 2,700 and 3,600 cells/mm^2^, depending on the area of mesh fibers contained in the ROIs. As expected, the foreign body reaction of the mesh–tissue complexes within the ROI presented a high proportion of innate immune cells, with 25% CD68^+^ macrophages, 20.2% CD11b^+^ myeloid cells, and 9.8% CD56^+^ NK cells (Table 3; Figs. 3–4). However, adaptive immune cells were also frequently observed, ranging from 5.2% for CD20^+^ B cells to 24.7% for FoxP3^+^ T_regs_. The mean CD4^+^/CD8^+^ ratio was 1.7 with a standard error of 0.5, indicating overall well-functioning patient immune systems (CD4^+^/CD8^+^ ratio > 1) [21].

**Table 3:** Analysis of innate and adaptive immune cells participating in the foreign body reaction to polypropylene meshes using regions of interest

Marker (*n* = 7 PP meshes)	Cell density (cells/mm^2^)		% Marker^+^/Nuclei		% Marker^+^CD68^−^/Marker^+^ “conventional”	
	Mean	SE	Mean	SE	Mean	SE
Adaptive						
CD3^+^	3,313.5	152.6	18.2	1.0	64.5	2.1
CD4^+^	3,584.1	137.5	19.2	1.3	38.1	2.2
CD8^+^	3,521.2	164.9	13.5	1.0	53.1	2.3
CD20^+^	3,184.1	105.5	5.2	0.9	71.3	3.8
FoxP3^+^	2,737.6	51.4	24.7	1.0	57.9	1.3
Innate						
CD11b^+^	3,065.7	102.5	20.2	1.5	52.0	2.1
CD56^+^	3,108.3	80.0	9.8	1.1	45.2	2.6
CD68^+^	3,206.1	51.6	25.0	0.5	–	–

The cell density is defined as the mean number of cells per mm^2^. Percentages of single-positive (Marker^+^) cells in relation to all cells and the proportions of “conventional” (Marker^+^CD68^−^) cells as function of all Marker^+^ cells. Hybrid cells with co-expression of CD68 were considered “disturbed”

### Innate and adaptive immune cells cluster toward the mesh fibers

**Fig. 5:**
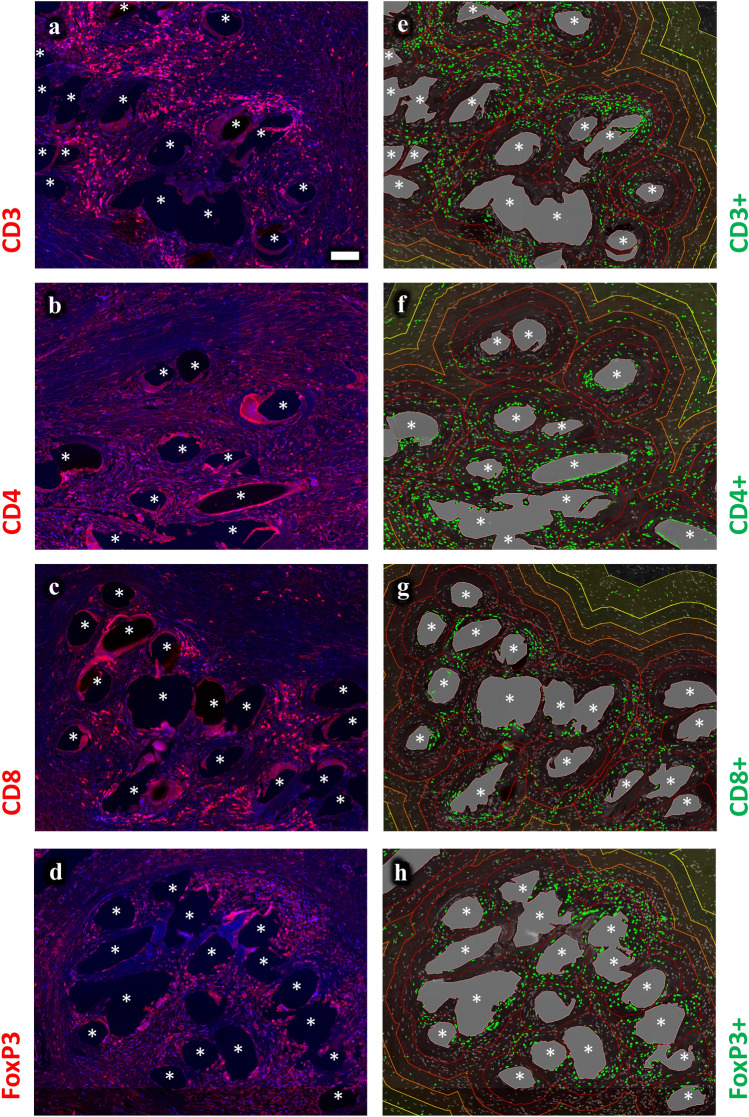
Spatial analysis of immunofluorescence labeled immune cells using Euclidean distance maps. Labeling of T cells (CD3), T_h_ (CD4), T_c_ (CD8), and T_regs_ (CD8). Nuclei are labeled with DAPI (blue). **a**–**d** Labeling of immune cells with Cy5 (red) and **e**–**h** backward gating of “positive” cells (green) with superimposed distance map, respectively. The Euclidean distance map consists of six regional zones from 0–50 µm (dark red) to 250–350 µm (bright yellow) in 50 µm steps. Locations of mesh fibers are marked with asterisks, scale bar = 100 µm. Images of the other cell types with distance maps are provided in the supplementary file (s-Fig. 5). Images of explant #7 (color figure online)

**Fig. 6:**
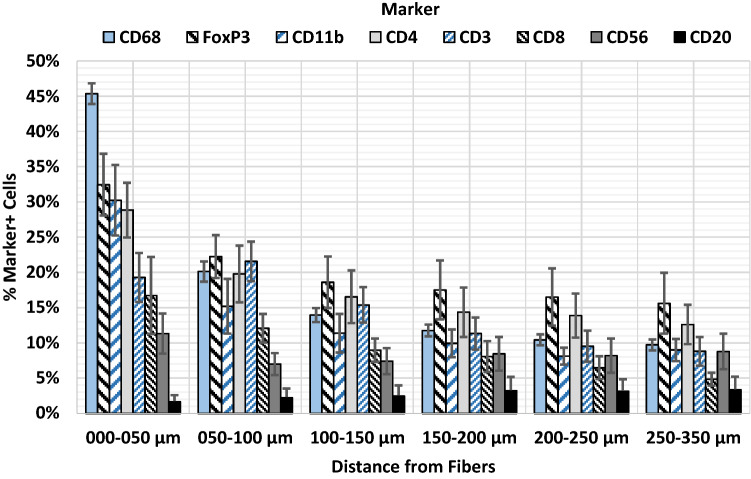
Spatial distribution of innate (CD68, CD11b, CD56) and adaptive (CD3, CD4, CD8, CD20, FoxP3) immune cells at the mesh–tissue interface. The bars represent the mean percentages of positive cells in each regional zone. Whiskers mark the SEs (*n* = 7 meshes). Cells with a mean staining intensity > 100 are considered to be “positive”. Innate immune cells are colored light blue and adaptive immune cells gray/black (color figure online)

**Fig. 7:**
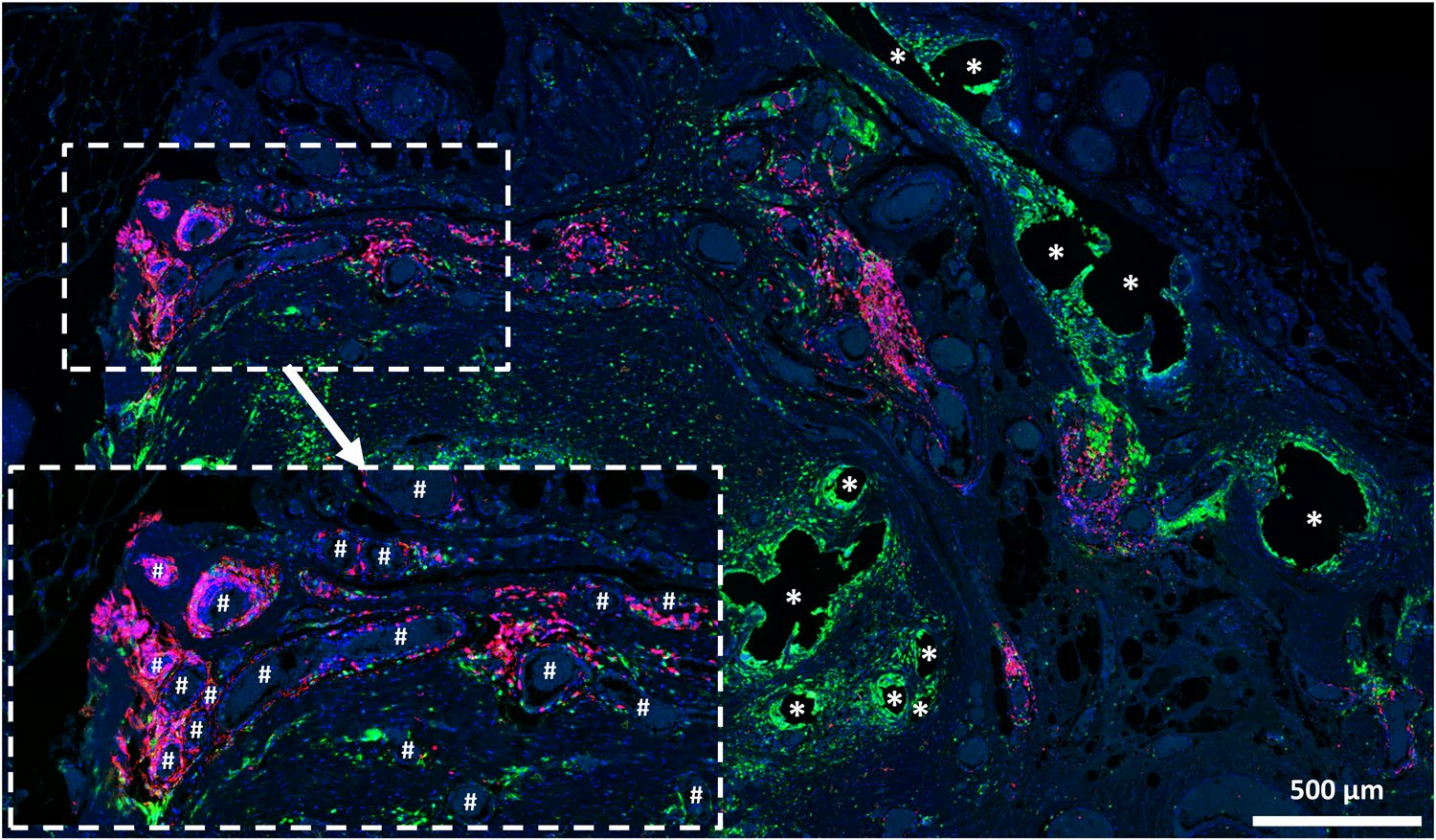
Dense clusters of B cells in the vicinity of the foreign body granuloma**.** Immunofluorescence labeling for macrophages (CD68, green) and B cells (CD20, red). Mesh fibers surrounded by macrophages with dense accumulations of B lymphocytes nearby, located around blood vessels (marked with ‘#’). Locations of mesh fibers are marked with asterisks. Image of explant #2 (color figure online)

Calculation of distance maps allowed to analyze the spatial distribution of the immune cells within the foreign body granuloma (FBG) (Fig. 5). In concordance with the previous data [20], the analysis revealed a significantly decreasing cell density and number of CD68^+^ macrophages with distance to the mesh fibers, from 4,418 cells/mm^2^ in zone1 (0–50 µm) to 1,696 cells/mm^2^ in zone6 (250–350 µm), and from 45.4 to 9.7%, respectively (Table 4; Fig. 6). For a rising distance up to 100 and 150 µm a decrease was also observed for CD56^+^ NK cells and CD11b^+^ myeloid cells. The highest frequencies of adaptive immune cells were observed in zone1 and zone2 (0–100 µm), except for CD20^+^ B cells, which were generally rare (Table 5; Fig. 6). Interestingly, however, dense clusters of B cells were frequently observed clustered around blood vessels near the FBG (Fig. 7). Except for B cells and NK cells, all investigated cell types decreased significantly from zone1 to zone6 (Tables 4, 5).

**Table 4:** Spatial analysis of innate immune cells and the proportions of “conventional” cells participating in the foreign body reaction using Euclidean distance maps

Parameter (*n* = 7 PP meshes)	Distance from mesh fibers					
	Zone1 000–050 µm	Zone2 050–100 µm	Zone3 100–150 µm	Zone4 150–200 µm	Zone5 200–250 µm	Zone6 250–350 µm
Cell density (cells/ mm^2^)	4,418.2 (80.2)	3,602.8 (98.6)^a^	2,849.3 (95.2)	2,386.1 (89.7)	2,018.9 (91.8)	1,696.1 (108.4)^b^
% CD11b^+^	30.2 (5.0)	15.2 (3.9)	11.4 (2.7)	9.9 (2.0)	8.1 (1.2)	9.0 (1.6)^b^
% CD11b^+^CD68^−^/CD11b^+^ “conventional”	37.2 (3.9)	63.9 (5.6)^a^	67.5 (5.4)	68.0 (6.3)	66.2 (6.2)	65.7 (4.8)^b^
% CD56^+^	11.3 (2.9)	7.0 (1.6)	7.4 (1.9)	8.4 (2.4)	8.2 (2.4)	8.8 (2.5)
% CD56^+^CD68^−^/CD56^+^ “conventional”	18.1 (2.9)	61.9 (9.5)^a^	73.4 (8.0)	75.1 (5.6)	78.2 (7.5)	83.3 (5.6)^b^
% CD68^+^	45.4 (1.5)	20.1 (1.4)^a^	14.0 (1.0)	11.8 (0.8)	10.4 (0.8)	9.7 (0.8)^b^

The cell density is defined as the mean number of cells per mm^2^. Percentages of single-positive (e.g., % CD56^+^) cells in relation to all cells and the proportions of “conventional” cells with respect to the total number of positive cells (e.g., % CD56^+^CD68^−^/CD56^+^). Hybrid cells with co-expression of CD68 were considered “disturbed”. The data are presented as mean (SE). Significant differences between zones according to Mann–Whitney *U* test: ^a^zone1 and zone2, ^b^zone1 and zone6

**Table 5:** Spatial analysis of adaptive immune cells and the proportions of “conventional” cells participating in the foreign body reaction using Euclidean distance maps

Parameter (*n* = 7 PP meshes)	Distance from mesh fibers					
	Zone1 000–050 µm	Zone2 050–100 µm	Zone3 100–150 µm	Zone4 150–200 µm	Zone5 200–250 µm	Zone6 250–350 µm
Cell density (cells/mm^2^)	4,418.2 (80.2)	3,602.8 (98.6)^a^	2,849.3 (95.2)	2,386.1 (89.7)	2,018.9 (91.8)	1,696.1 (108.4)^b^
% CD3^+^	19.3 (3.5)	21.6 (2.8)	15.4 (2.5)	11.3 (2.3)	9.5 (2.2)	8.8 (2.0)^b^
% CD3^+^CD68^−^/CD3^+^ “conventional”	47.8 (9.1)	70.8 (8.4)	73.3 (8.1)	75.0 (6.3)	73.0 (7.0)	70.3 (6.0)
% CD4^+^	28.8 (3.9)	19.8 (4.0)	16.5 (3.7)	14.4 (3.5)	13.9 (3.1)	12.6 (2.8)^b^
% CD4^+^CD68^−^/CD4^+^ “conventional”	20.7 (4.1)	50.5 (7.9)^a^	54.6 (6.5)	56.2 (5.6)	58.4 (5.5)	57.5 (7.4)^b^
% CD8^+^	16.7 (5.5)	12.1 (2.0)	9.0 (1.7)	8.1 (2.2)	6.5 (1.6)	4.8 (0.9)^b^
% CD8^+^CD68^−^/CD8^+^ “conventional”	44.0 (8.1)	79.3 (4.2)^a^	83.6 (3.4)	78.3 (4.6)	72.2 (4.9)	77.5 (4.4)^b^
% CD20^+^	1.7 (0.9)	2.2 (1.3)	2.5 (1.5)	3.2 (2.0)	3.1 (1.7)	3.3 (1.9)
% CD20^+^CD68^−^/CD20^+^ “conventional”	57.3 (12.1)	91.8 (6.8)	92.4 (3.0)	95.3 (2.8)	85.2 (10.8)	95.1 (2.5)
% FoxP3^+^	32.4 (4.4)	22.2 (3.1)	18.6 (3.6)	17.5 (4.2)	16.5 (4.1)	15.6 (4.3)^b^
% FoxP3^+^CD68^−^/FoxP3^+^ “conventional”	33.5 (1.5)	67.6 (3.9)^a^	79.9 (3.4)	83.7 (3.3)	87.3 (2.0)	90.7 (2.2)^b^

The cell density is defined as the mean number of cells per mm^2^. Percentages of single-positive (e.g., % CD3^+^) cells in relation to all cells and the proportions of “conventional” cells with respect to the total number of positive cells (e.g., % CD3^+^CD68^−^/CD3^+^). Hybrid cells with co-expression of CD68 were considered “disturbed”. The data are presented as mean (SE). Significant differences between zones according to Mann–Whitney *U* test: ^a^zone1 and zone2, ^b^zone1 and zone6

### Proportions of “conventional” immune cells increase with distance to the mesh fibers

**Fig. 8:**
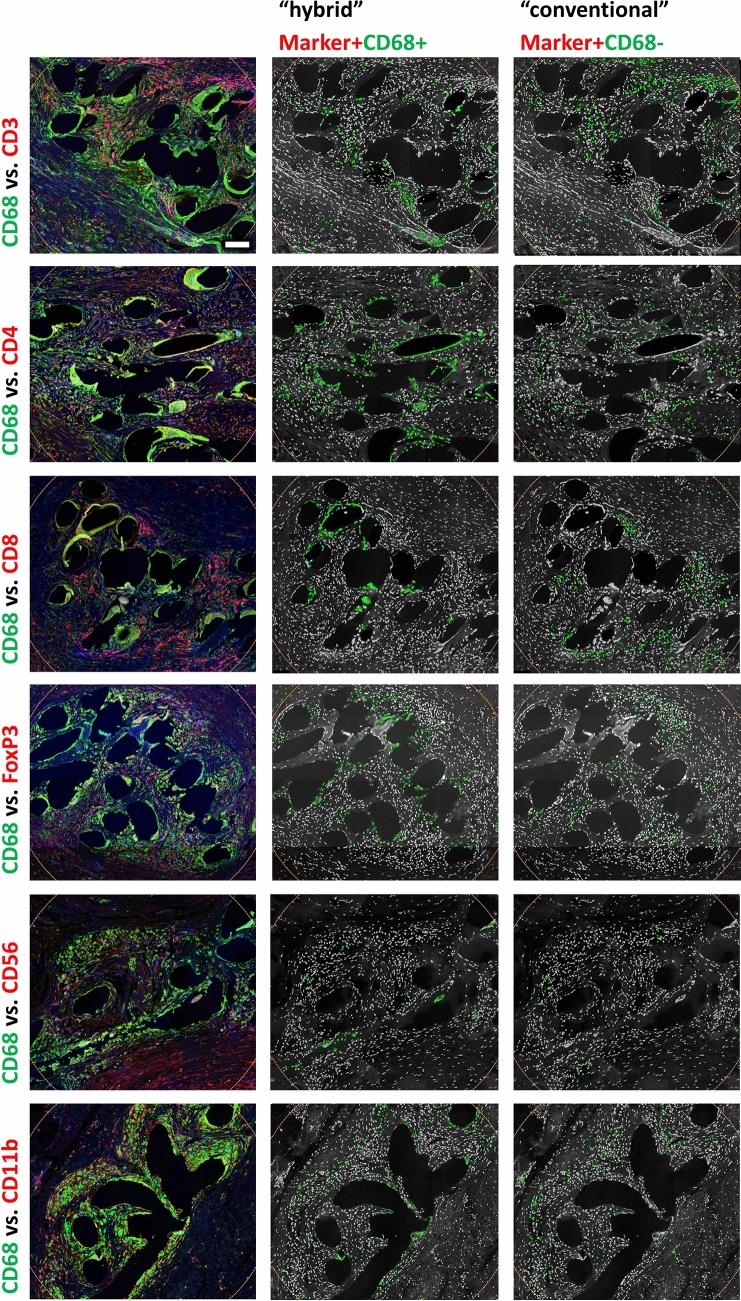
“Hybrid” and “conventional” immune cells. **1. column** Double stainings for macrophages (CD68) with FITC (green) and T cells (CD3), T_h_ (CD4), T_c_ (CD8), T_regs_ (FoxP3), NK cells (CD56), or myeloid cells (CD11b) with Cy5 (red). **2. column** Backward gating of “hybrid” immune cells (green) and **3. column** “Conventional” immune cells (green) on grayscale nuclei shades, respectively. Cells with a mean staining intensity > 100 are considered to be “positive”, scale bar = 100 µm (color figure online)

Previously, we reported that in the area of the granuloma CD68 is co-expressed on various cell types participating in the FBR, which is considered a “hybrid” pattern due to the sustained mesh-induced stimulus [19]. This co-expression is rarely present (< 5%) in control tissues (s-Table 1). In this study, we confirmed the abundant co-expression of CD68 in various types of immune cells (Fig. 8). To investigate at what distance the immune cells transition toward a more physiological “conventional” state marked by low CD68 co-expression, we looked for the fraction of the total number of positive cells for each cell type not co-expressing CD68, e.g., CD4^+^CD68^−^/CD4^+^ for T_h_, in relation to the distance of the mesh fibers.

**Fig. 9:**
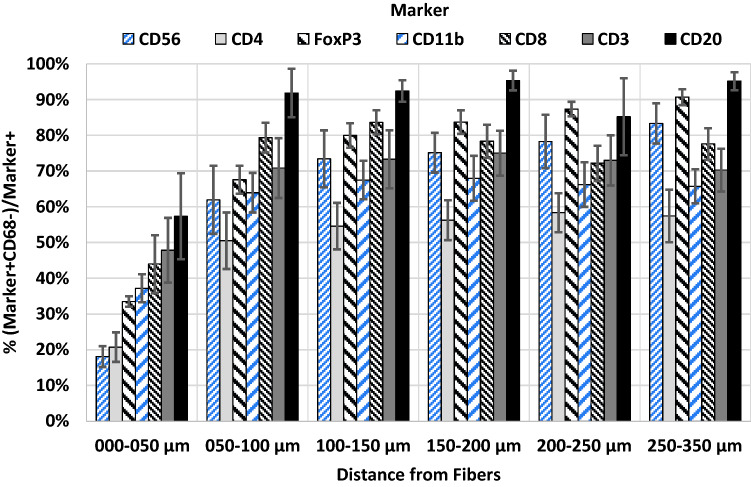
Spatial characterization of “conventional” cells. Proportions of “conventional” cells (defined as cells not co-expressing CD68) in relation to the total number of positive cells (e.g., CD4^+^CD68^−^/CD4^+^ for T-helper cells). The bars represent the mean proportions in each regional zone. Whiskers mark the SEs (*n* = 7 meshes). Cells with a mean staining intensity > 100 are considered to be “positive”. Innate immune cells are colored light blue and adaptive immune cells gray/black (color figure online)

Overall, within the FBG, CD4^+^ T_h_ presented the lowest proportion of “conventional” cells with 38.1%, while CD20^+^ B cells presented the highest with 71.3% (Table 3). Spatial analysis with distance maps revealed for all investigated cell types the lowest proportion of “conventional” cells in zone1 (0–50 µm) directly at the mesh–tissue interface, with the lowest proportion for CD56^+^ NK cells (18.1%) and the highest for CD20^+^ B cells (57.3%) (Tables 4, 5; Fig. 9). From zone1 to zone2 (50–100 µm), the proportion of “conventional” cells increased for all cell types, but being significant only for CD4^+^ T_h_, CD8^+^ T_c_, FoxP3^+^ T_regs_, CD11b^+^ myeloid cells, and CD56^+^ NK cells. Noteworthy, from zone2 to zone6 (250–350 µm), the proportions remained relatively constant except FoxP3^+^ T_regs_ and CD56^+^ NK cells, whose proportions increased steadily with distance to the mesh fibers (Tables 4, 5).

## Discussion

The study of innate and adaptive immune cells in human polypropylene (PP) mesh explants from the abdomen clearly demonstrates that cells from both arms of the immune system participate in the foreign body reaction (FBR) after implantation.

Lymphocytes, such as T_h_ and T_regs_ were present at comparable rates to macrophages (25%) with 19.2% and 24.7%, respectively, demonstrating that both innate and adaptive cells are likely to collaborate in the foreign body granuloma (FBG). Furthermore, all investigated immune cell types significantly accumulate in vicinity of the mesh fibers, except for NK and B cells. Irrespective of individual clustering, all cell types at the interface (0–50 µm) showed “disturbance” with co-expression of CD68; whereas cells transitioned to a more physiological “conventional” state at around 50–100 µm, demonstrating the successful shielding by the fibrotic capsule at the outer limitation of the FBG.

All specimens presented a pronounced FBR, which was restricted to the vicinity of the mesh fibers consisting of dense cellular infiltrates of mostly mononuclear cells. Using circular ROIs placed around mesh fibers, macrophages and T cells were found to be the predominant cell types within the FBG, which is in line with other studies on PP meshes [16, 17, 22, 23]. T_regs_ and T_h_ were the most frequent T subsets with 25% and 19.2%, respectively, whereas T_c_ were found at a lower rate of 13.5%. The finding of more T_h_ than T_c_ is consistent with Tennyson et al. [16]. In contrast to our results, Artsen et al. and Tennyson et al. observed fewer T_regs_ than T_c_ in human PP mesh explants from the pelvic floor removed for pain or exposure [16, 17]. This difference may be explained by the intrinsic difficulty to quantify immunohistochemistry at little standardized samples. Quantification strongly depends on markers, the cutoff, and the location and size of the investigated region. Not least, the medical reason for mesh removal may interfere. Furthermore, there are likely differences in host reactions considering different anatomical locations, such as pelvic floor and abdominal wall [20, 24, 25].

The role of FoxP3^+^ T_regs_ in the FBR is still controversial. It has been suggested that they promote a fibrotic response on the one hand and have an antifibrotic effect on the other [16, 17]. In general, T_regs_ are known for their role in maintaining immune homeostasis via secretion of anti-inflammatory cytokines, expression of co-inhibitory molecules, modulation of antigen-presenting cells, and depletion of growth factors from the microenvironment [26]. Furthermore, they are capable of mediating several extra-immune functions, such as angiogenesis and tissue repair [26]. FoxP3 expression can be induced in various T cells, and has been reported in NK cells and macrophages, too [26]. With regards to the latter, the co-expression with CD68 in over 40% of FoxP3^+^ cells may indicate a potential role of FoxP3 in mesh-associated activation of macrophages. Furthermore, in light of the controversy whether T_regs_ promote or reduce fibrosis, the high level of FoxP3^+^ cells observed here, in combination with the previous studies of the same specimens that clearly showed excessive collagen deposition [20], may suggest T_regs_ are not antifibrotic in our samples.

Within the FBGs, CD20^+^ B cells were rare at about 5%, which is in agreement with several studies [4, 23, 27], but interestingly dense clusters were frequently found near the FBGs accumulated around blood vessels. This finding may gain importance by the fact that B cells influence T cell responses and affect fibrosis through interactions with other cells including fibroblasts and macrophages [28]. Furthermore, proliferation of B cells in extramedullary environments allows for survival of self-reactive B cells, thereby increasing the potential for autoimmune pathology [29].

Another marker that has been studied is CD11b (also referred to as macrophage receptor 1 ‘Mac-1’), which is known for its expression on myeloid cells. CD11b mediates cellular adhesion, migration, and activation, and therefore plays a central role in inflammation [30]. It generally modulates pro- and anti-inflammatory signaling in cells [31]. As has been shown in human and murine cancers studies, tumor associated macrophages primarily exhibit an immunosuppressive/anti-inflammatory phenotype that significantly correlated with the expression of CD11b [32, 33]. The high number of CD11b^+^ cells (20.2%) observed in our samples, with half of them co-expressing CD68, underline the involvement of activated innate immune cells in the FBR. The considerable expression of CD11b indicates a rather anti-inflammatory function in this location, and corresponds with our previous data [20], where we have identified most macrophages within the granuloma as anti-inflammatory type expressing CD206 and CD163.

Spatial analysis using distance maps revealed that the proportions of positive cells for all immune cell types studied were highest in zone1 (0–50 µm) or zone2 (50–100 µm), except for CD20 B cells. Consequently, these zones also contained the highest levels of CD68 co-expression and thus the lowest proportions of “conventional” cells. Most of these cells co-expressing CD68 are probably macrophages known for their remarkable plasticity that allows them to efficiently respond to environmental signals and change their phenotype and physiology [34]. In fact, previous studies have reported that macrophages can co-express several markers usually linked to other immune cells, such as CD3 [35–38], CD4 [39–42], CD8 [39, 41, 43], and FoxP3 [44]. Vice versa, other innate immune cell types, such as NK cells [45, 46] and adaptive immune cells, such as lymphocytes [46, 47] have been shown to express CD68. The observed co-expression of CD68 with lymphoid markers in cells governing the immune response indicate an unconventional activation/“disturbance” of cells due to the persistent stimulus by the meshes. This change of the functional landscape of the involved cells is similar to effects observed in infectious/inflammatory and tumor pathologies [35, 45, 48]. However, to decipher the underlying mechanisms of this heterogeneity, more thorough investigations using methods of extended multiplex stainings as well as lineage tracing are required.

For further spatially investigation of the FBR and to characterize the mesh-induced stimulus, we determined the proportions of “conventional” immune cells with expression of their specific immune cell marker and without co-expression of CD68 in each distance zone. In line with the high level of CD68 co-expression at the mesh–tissue interface, the proportions of “conventional” cells were lowest in zone1, but increased substantially in zone2 for all cell types; and remained relatively constant thereafter. The highest percentages of innate CD68^+^ macrophages and innate “conventional” CD11b^+^CD68^−^ myeloid cells were found directly at the mesh–tissue interface (0–50 µm), whilst highest percentages of “conventional” adaptive immune cells were found at greater distances. In addition, a significant decrease of cell density was observed from zone1 to zone3 (0–150 µm). Taken together, these results illustrate the transition from dense cellular infiltrate to collagen-rich fibrotic capsule at about 50–100 µm from the mesh fibers, and the attenuation of the mesh-induced stimulus by the fibrotic capsule, allowing most cells to maintain a “conventional” state. Several animal studies have investigated the size of the cellular infiltrate in relation to PP meshes [49–53]. The size of the cellular infiltrate obtained here with approximately 50 µm diverges from data of these studies (9–35 µm). There are several reasons for this. Amongst others, first, animal studies either lack a naturally occurring abdominal wall defect or an artificial defect in an otherwise intact abdominal wall is created. Second, there are inherent differences in the anatomy and meshes are not adapted to the physiology of animals. Finally, the size of the infiltrate strongly depends on the type of evaluation.

Based on the distributions of “conventional” cells and the cellular composition of the FBG with innate immune cells predominantly located centrally at the interface and adaptive immune cells peripheral, there seems to be some overlap with other granulomas, such as observed, for example, in sarcoidosis [10]. However, although the FBG of mesh always includes a fibrotic capsule, this is not always the case with sarcoidosis and foremost etiologies are different. Nevertheless, the FBG of mesh may serve as model to study the highly complex and coordinate interplay of the many diverse immune cell populations involved in granulomas. Interestingly, in the FBR of mesh, mainly innate immunity is studied as opposed to granulomatous inflammatory diseases. This is probably due to the natural time course of the FBR, the (early) observed predominance of macrophages, and time limit constraints in animal experiments.

Since the FBR is obviously an ongoing chronic process that never completely stops, risks may accumulate over the years, warranting caution of mesh utilization in younger patients. Given a sustained immunological response with abundant embedded T cells and B cell clusters questions its possible role in autoimmune diseases. As Cohen Terveart has postulated, patients may be at risk of developing (auto)immune disease when PP mesh is utilized; particularly, with pre-existing allergic disease [18]. Considering these facts, clearly, future attempts to enhance biocompatibility of meshes also need to consider the adaptive immunity.

Several limitations of our study have to be considered. The collection of explanted meshes comprised three different PP meshes with different textile properties (e.g., mechanical strength, pore sizes). Although the PTFE layer of the Ventralex^®^ meshes has been spatially excluded with adjacent tissues, we cannot completely rule out any far-distant influence on the wound area. Furthermore, the selection of patients only included patients with recurrence of abdominal wall hernia after varying incorporation time. In addition to many possible confounders introduced by the complex staining protocol, the location and size of the investigated region will influence the results, as well. To compensate the latter, we used standardized 1 mm^2^ circular regions of interest (ROIs) that were placed around mesh fibers. In addition, we also used a Euclidean distance map algorithm that created six regional zones adjacent to the mesh fibers, allowing for spatially discrete quantification. Our choice of 1 mm^2^ ROIs and six regional zones covering 350 µm from the mesh interface ensured that most of the FBG was included. By relating the number of positive cells to the total number of cells we created a robust, normalized measurement. For cells, the analysis of staining intensity only in the nucleus area cannot exclude positive staining based on some overlapping cytoplasmic membrane of adjacent cells, but the probability is considerably reduced by a cut thickness of 2 µm.

We applied a high cutoff value of 100 for the mean staining intensity of cells, as described [19], which allowed us to determine positive cells objectively and reliably and gives us the highest confidence for true positive detection. However, the consequence of this is that cells considered as positive need to have a high staining intensity, resulting in lower rates than would have been seen with lower cutoffs.

In conclusion, the findings of the present study clearly show that both the innate and the adaptive immune system participate in the long-term foreign body reaction to polypropylene meshes. T cells of the adaptive immune system were found with similar rates as macrophages. However, in concordance with the previous data [19], many cells at the mesh–tissue interface presented a “hybrid” pattern, as evidenced by signs of cross-lineage heterogeneity. Future attempts to enhance biocompatibility of meshes need to consider the adaptive immunity, focus on a reduction of the heterogeneous inflammatory infiltrate and the promotion of better wound healing.

## Supplementary Information

Below is the link to the electronic supplementary material.

Supplementary file1 (PDF 1085 KB)

## Data Availability

The raw data required to reproduce these findings are available on request from the authors.
