# Supplementary material for: Characterization of innate and adaptive immune cells involved in the foreign body reaction to polypropylene meshes in the human abdomen
Source: Hernia. 2021 Mar 31;26(1):309–23. doi: 10.1007/s10029-021-02396-7 (PMC8881270; doi:10.1007/s10029-021-02396-7)
Supplement: Supplementary file 1 — Supplementary file1 (PDF 1085 KB) [file 10029_2021_2396_MOESM1_ESM.pdf]

# Characterization of innate and adaptive immune cells involved in the foreign body reaction to polypropylene meshes in the human abdomen

*Journal* HERNIA

*Authors* *Axel Dievernich[1], Pascal Achenbach[2], Luke Davies[3], Uwe Klinge[1]

[1] Department of General, Visceral and Transplant Surgery, RWTH Aachen University Hospital, Aachen, Germany

[2] Institute of Neuropathology, RWTH Aachen University Hospital, Aachen, Germany

[3] Division of Infection and Immunity, Cardiff University, Cardiff, UK

* Corresponding author

A. Dievernich

Department of General, Visceral and Transplant Surgery, RWTH Aachen University Hospital, Pauwelsstraße 30, 52074 Aachen, Germany

e-mail: adievernich@web.de

**s-Fig. 1** Restriction of analysis and exclusion of the PTFE layer from analysis. **a** Immunofluorescence labeling of macrophages (CD68) with FITC (green) and T cells (CD3) with Cy5 (red). **b** To minimize the influence of the PTFE layer, the analysis was restricted (solid orange line) and cells near the layer were excluded (purple exclusion area). Dashed orange, white lines mark manually selected mesh fiber areas, which were used as a basis for the Euclidean distance map algorithm. Locations of mesh fibers are marked with asterisks

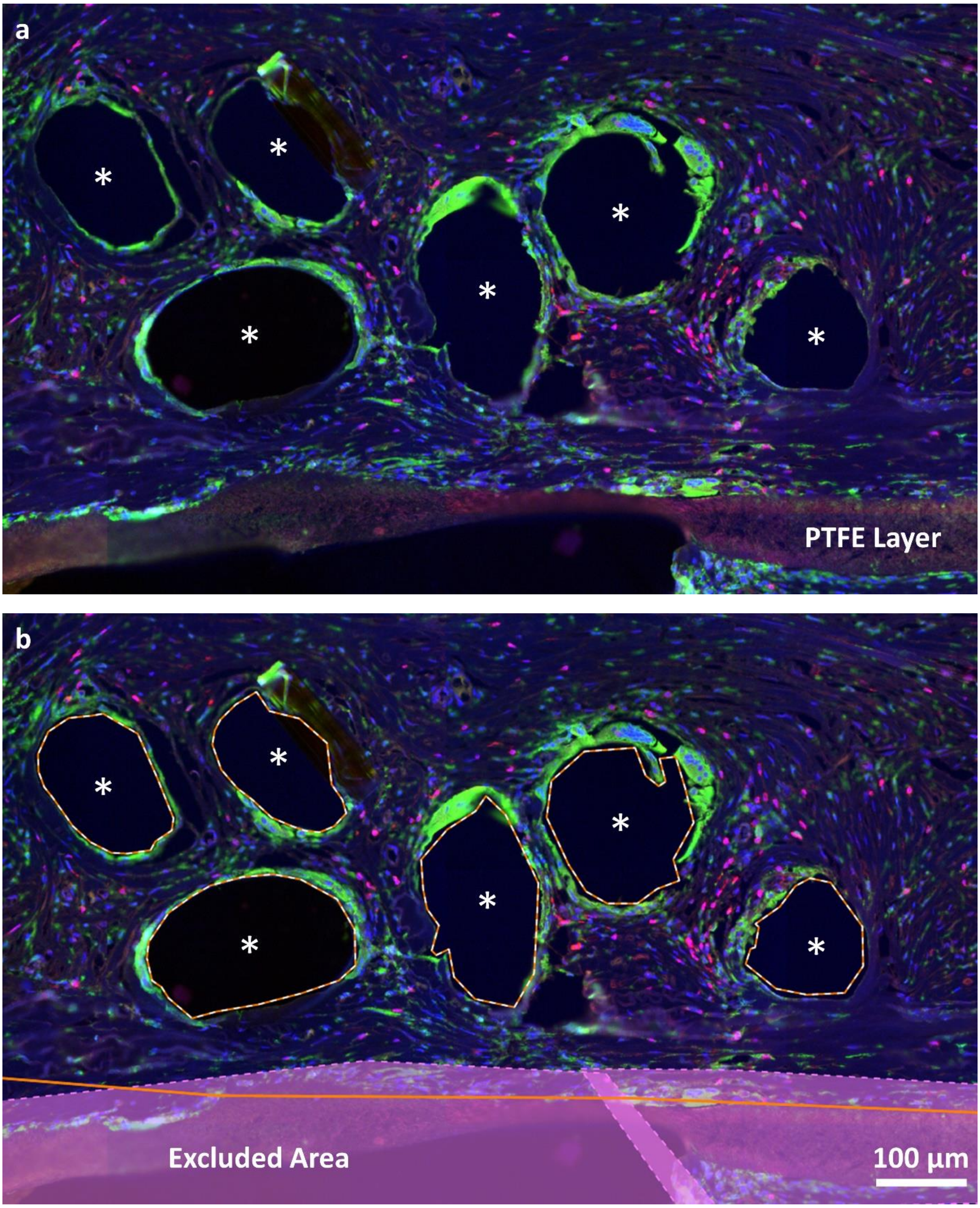

**s-Fig. 2** Immunofluorescence labeling of CD68 (FITC, green), CD8 (Cy5, red), and nuclei (DAPI, blue) in human tonsil tissue

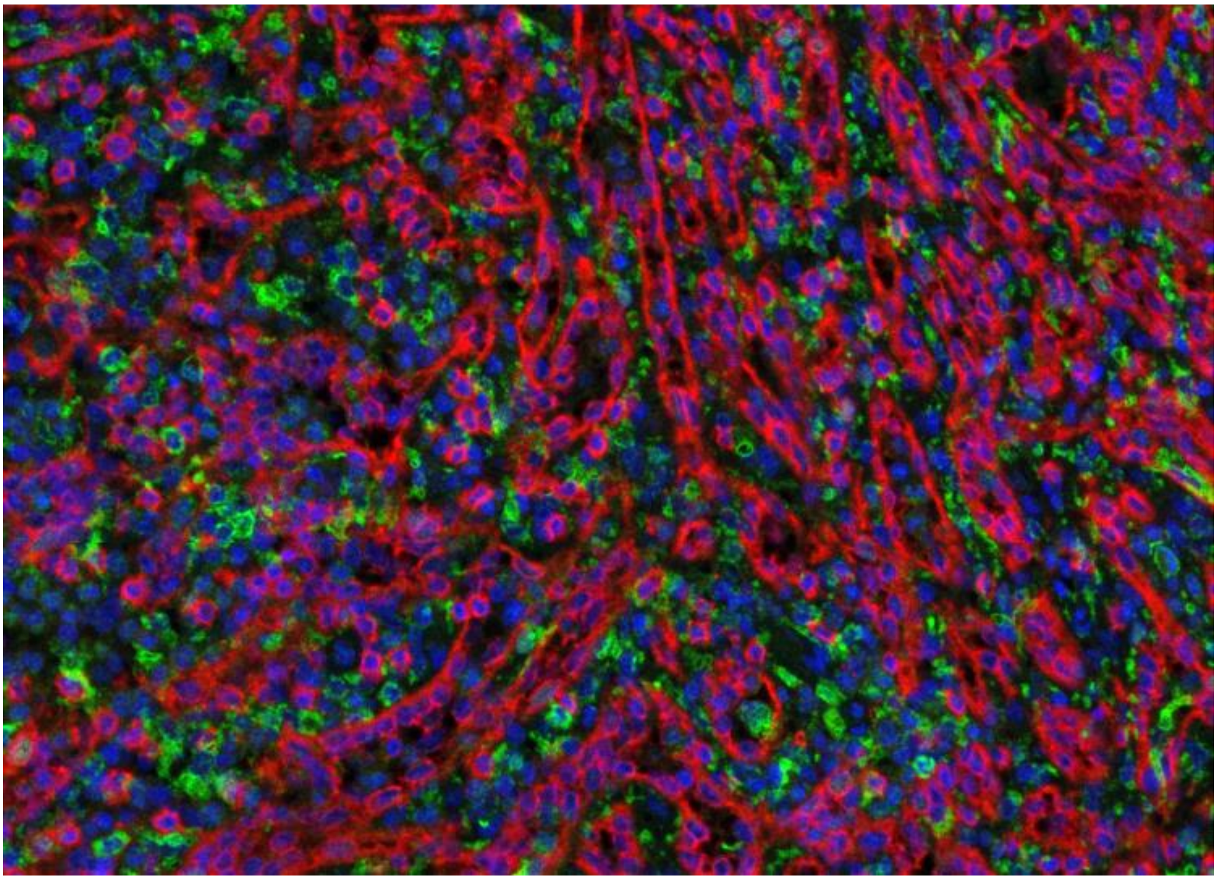

**s-Fig. 3** Immunofluorescence labeling of CD68 (FITC, green), CD4 (Cy5, red), and nuclei (DAPI, blue) in human tonsil tissue.

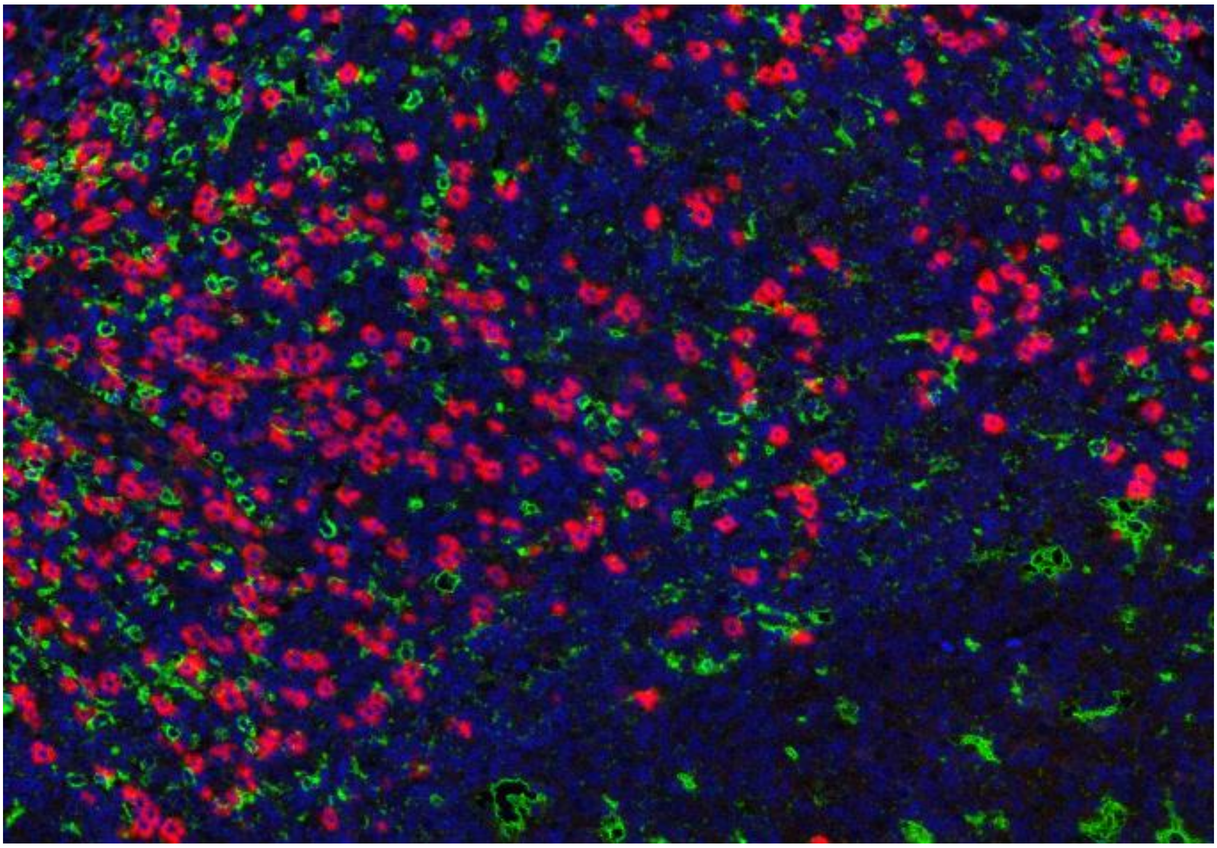

**s-Fig. 4** Immunofluorescence labeling of CD68 (FITC, green), CD3 (Cy5, red), and nuclei (DAPI, blue) in human lymph node tissue.

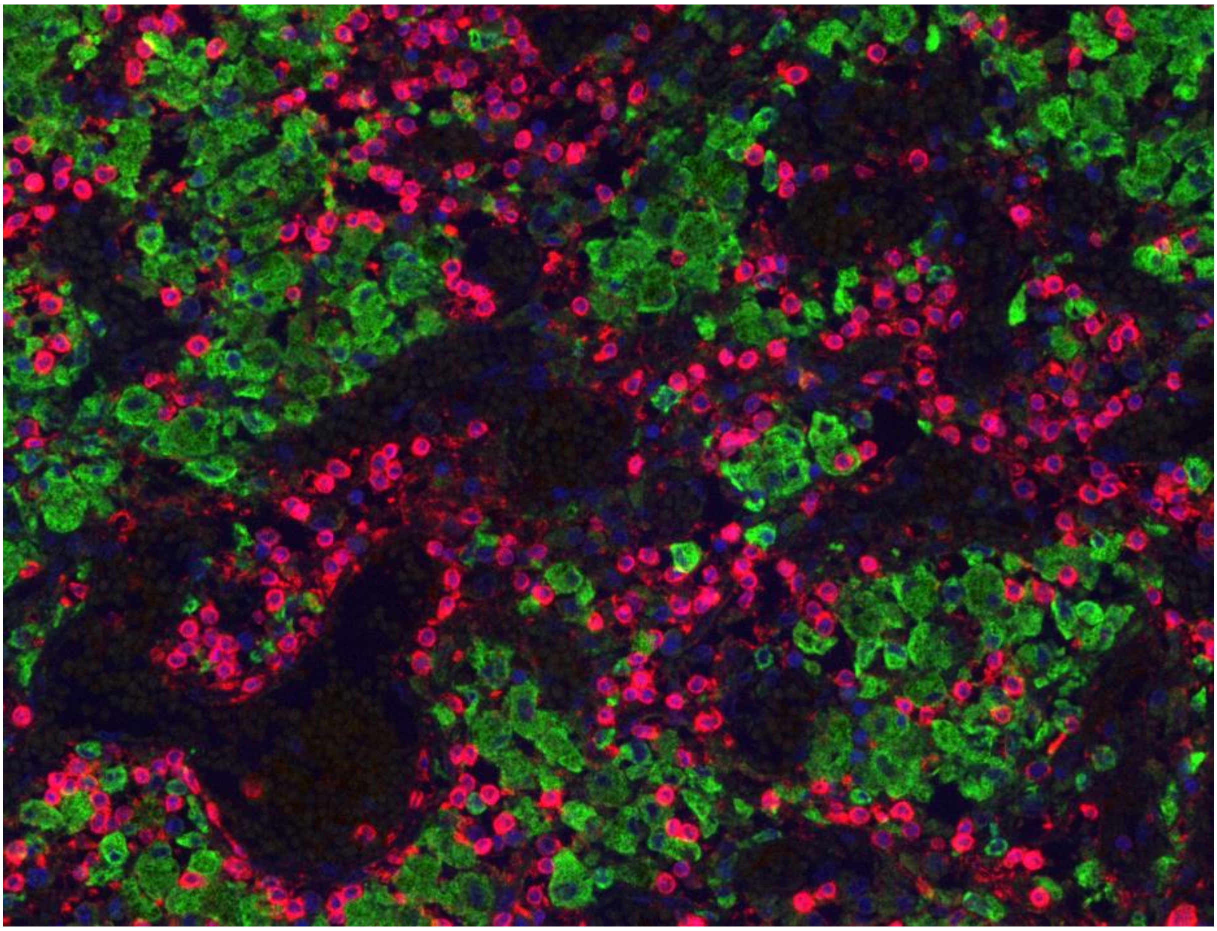

**s-Fig. 5** Spatial analysis of immunofluorescence labeled immune cells using Euclidean distance maps. Labeling of B cells (CD20), NK cells (CD56), and myeloid cells (CD11b). Nuclei are labeled with DAPI (blue). **a-c** Labeling of immune cells with Cy5 (red) and **e-g** backward gating of “positive” cells (green) with superimposed distance map, respectively. The Euclidean distance map consists of six regional zones from 0-50 µm (dark red) to 250-350 µm (bright yellow) in 50 µm steps. Locations of mesh fibers are marked with asterisks. Images of the other cell types/ markers are provided in the supplementary file. **a, e** Images of explant #6, **b, f** images of explant #2, **c, g** images of explant #3

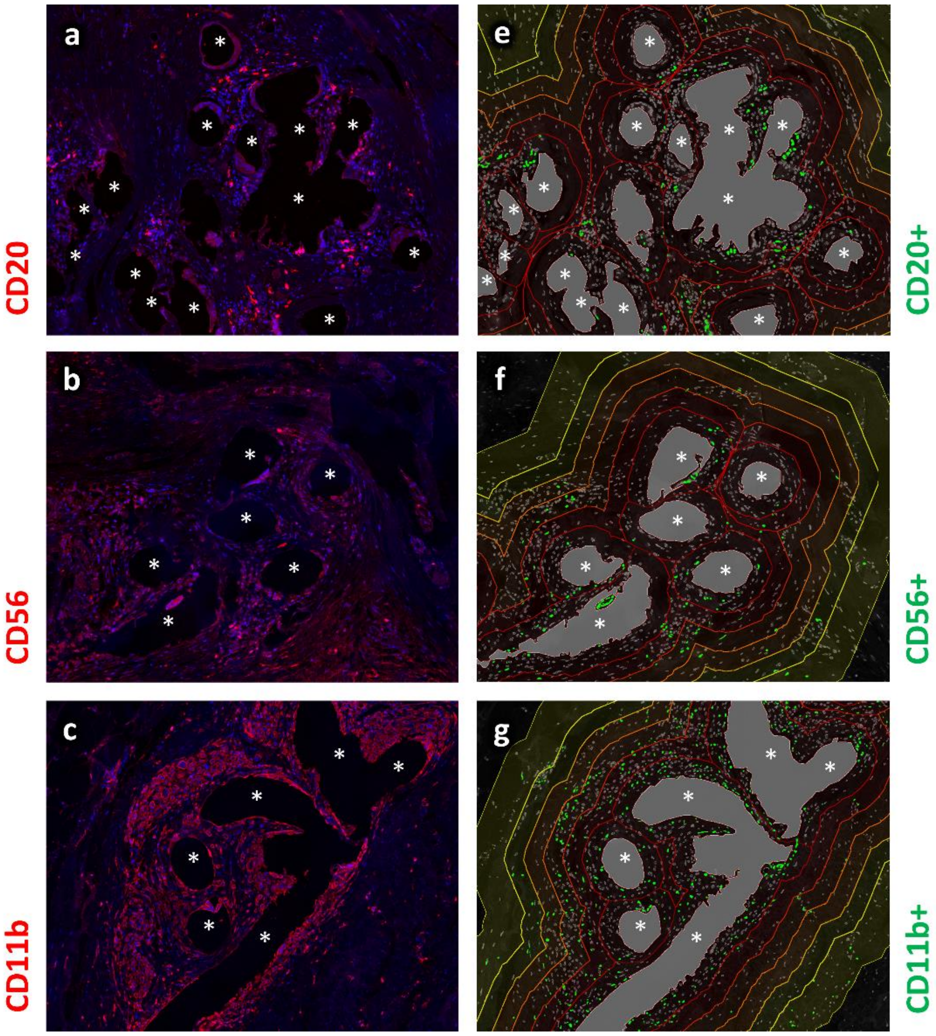

**s-Table 1** Analysis of adaptive and innate immune cells in human control tissue samples. The cell density is defined as the average number of cells per mm². Percentages of single-positive (Marker$^+$) cells in relation to all cells and the proportions of "unperturbed" (Marker$^+$CD68$^-$) cells as function of all Marker$^+$ cells. Co-expression with CD68 is considered a perturbation

| **Marker** | **Tissue** | **Cell density (cells/ mm²)** | | **% Marker$^+$/Nuclei** | **% Marker$^+$CD68$^-$/ Marker$^+$ "unperturbed"** |
|---|---|---|---|---|---|
| | | *min* | *max* | *mean* | *mean* |
| *CD3* | Liver | 5,712 | 7,018 | 2.6 | 100.0 |
| | Lymph Node | 6,554 | 9,545 | 32.4 | 99.6 |
| | Spleen | 10,044 | 12,987 | 13.5 | 98.4 |
| | Tonsil | 12,063 | 16,745 | 26.3 | 98.4 |
| *CD4* | Liver | 5,712 | 7,018 | 4.7 | 86.6 |
| | Lymph Node | 6,554 | 9,545 | 23.7 | 96.5 |
| | Spleen | 10,044 | 12,987 | 13.6 | 80.4 |
| | Tonsil | 12,063 | 16,745 | 5.3 | 99.9 |
| *CD8* | Liver | 5,712 | 7,018 | 1.7 | 97.6 |
| | Lymph Node | 6,554 | 9,545 | 6.4 | 100.0 |
| | Spleen | 10,044 | 12,987 | 31.9 | 99.7 |
| | Tonsil | 12,063 | 16,745 | 12.8 | 99.7 |
| *CD11b* | Liver | 5,712 | 7,018 | - | - |
| | Lymph Node | 6,554 | 9,545 | - | - |
| | Spleen | 10,044 | 12,987 | 12.3 | 94.1 |
| | Tonsil | 12,063 | 16,745 | 11.9 | 96.0 |
| *CD56* | Liver | 5,712 | 7,018 | - | - |
| | Lymph Node | 6,554 | 9,545 | - | - |
| | Spleen | 10,044 | 12,987 | 4.2 | 97.6 |
| | Tonsil | 12,063 | 16,745 | 0.4 | 100.0 |
| CD68 | Liver | 5,712 | 7,018 | 1.9-2.7 | - |
| | Lymph Node | 6,554 | 9,545 | 5.7-7.6 | - |
| | Spleen | 10,044 | 12,987 | 3.6-8.1 | - |
| | Tonsil | 12,063 | 16,745 | 1.9-2.7 | - |
